# Supplementary material for: Dynamic transcriptomic profiles of zebrafish gills in response to zinc supplementation
Source: BMC Genomics. 2010 Oct 11;11:553. doi: 10.1186/1471-2164-11-553 (PMC3091702; doi:10.1186/1471-2164-11-553)
Supplement: Additional file 2 — Interactive Direct Interaction Network representing the molecular interactions between zinc, copper, iron, calcium and proteins encoded by transcripts changed by zinc supplementation. Mini web-site containing index.html and hyperlinked pages in subdirectory describing a Direct Interaction Network automatically generated based on curated interactions contained within the proprietary PathwayArchitect database. Ovals represent proteins and the circles symbolize metal ions. Objects are coloured by their abundance in zebrafish at the time-point they were significantly different from the control is a scale from -4 fold (dark green) to +4 fold (dark red). Where significant differences were found at more than one time-point, the colour overlay shows expression at the first instance. Dark blue squares denote 'binding', and light blue squares 'expression'; green squares stand for 'regulation', green diamonds for 'metabolism', and green circles for 'promoter binding'. Arrow heads indicate directionality of the interaction where annotated. All nodes and edges can be further interrogated by selecting the relative area of the image. [file 1471-2164-11-553-S2.zip › PathwayArchitect Zn xs DIN/97189.html]

# PROTEIN: PTCH

|  |  |
| --- | --- |
| Name | PTCH |
| Type | PROTEIN |
| Description | patched homolog (Drosophila) |
| Note | This gene encodes a member of the patched gene family. The encoded protein is the receptor for sonic hedgehog, a secreted molecule implicated in the formation of embryonic structures and in tumorigenesis. This gene functions as a tumor suppressor. Mutations of this gene have been associated with nevoid basal cell carcinoma syndrome, esophageal squamous cell carcinoma, trichoepitheliomas, transitional cell carcinomas of the bladder, as well as holoprosencephaly. Alternative spliced variants have been described, but their full length sequences have not be determined. |
| Alias | PTCH1 |
|  | PTC1 |
|  | NBCCS |
|  | patched |
|  | PTC |
|  | patched (Drosophila) homolog |
|  | HPE7 |
|  | BCNS |
|  | PTCH |


---

|  |  |
| --- | --- |
| GO Component | integral to plasma membrane |
|  | membrane |


---

|  |  |
| --- | --- |
| GO ID | GO:0016020 |
|  | GO:0009653 |
|  | GO:0007165 |
|  | GO:0008283 |
|  | GO:0008158 |
|  | GO:0005887 |
|  | GO:0045786 |
|  | GO:0004872 |
|  | GO:0007049 |


---

|  |  |
| --- | --- |
| MIM | MIM:601309 |
|  | MIM:605462 |
|  | MIM:109400 |


---

|  |  |
| --- | --- |
| Connectivity | 78 |


---

|  |  |
| --- | --- |
| Entrez ID | 5727 |


---

|  |  |
| --- | --- |
| Agilent ID | A\_24\_P115490 |


---

|  |  |
| --- | --- |
| Cellular Localization | Membrane |
|  | Cell |
|  | Plasma membrane |


---

|  |  |
| --- | --- |
| DbXref | KEGG pathway##04340##Hedgehog signaling pathway##http://www.genome.jp/dbget-bin/show\_pathway?hsa04340+5727 |


---

|  |  |
| --- | --- |
| Pathway | Hedgehog Signaling |
|  | Zn xs inventory |
|  | Zn xs DIN |


---

|  |  |
| --- | --- |
| GO Process | cell proliferation |
|  | signal transduction |
|  | negative regulation of progression through cell cycle |
|  | cell cycle |
|  | morphogenesis |


---

|  |  |
| --- | --- |
| UniGene | Hs.494538 |


---

|  |  |
| --- | --- |
| Affymetrix Probeset ID | 1555520\_at |
|  | 208522\_s\_at |
|  | 209815\_at |
|  | 209816\_at |
|  | 238754\_at |
|  | 38214\_at |
|  | 59969\_at |
|  | 81835\_at |
|  | 836\_at |
|  | 90226\_at |
|  | g4506246\_3p\_a\_at |
|  | Hs.125230.0.A1\_3p\_at |
|  | Hs.159526.0.S2\_3p\_at |
|  | Hs.159526.0.S3\_3p\_at |
|  | Hs2Affx.1.349.S1\_3p\_at |
|  | U43148\_at |
|  | AFFX-hum\_alu\_at |
|  | hum\_alu\_at |


---

|  |  |
| --- | --- |
| GO Function | receptor activity |
|  | hedgehog receptor activity |


---

|  |  |
| --- | --- |
| Nucleotide | NM\_000264 |
|  | AB189438 |
|  | U43148 |
|  | AB209495 |
|  | AY395758 |
|  | AY395772 |
|  | AB189439 |
|  | AI494442 |
|  | BC043542 |
|  | BM974119 |
|  | AB189440 |
|  | AB189437 |
|  | U59464 |
|  | AB189436 |
|  | AY395768 |
|  | AL161729 |


---

|  |  |
| --- | --- |
| Protein | AAC50496 |
|  | AAH43542 |
|  | AAC50550 |
|  | BAD74187 |
|  | AAR21240 |
|  | BAD74184 |
|  | NP\_000255 |
|  | CAH73818 |
|  | BAD74186 |
|  | Q13635 |
|  | BAD74185 |
|  | AAR21238 |
|  | BAD92732 |
|  | BAD74188 |
|  | AAR21239 |
|  | CAH73817 |


---

|  |  |
| --- | --- |
| Organism | Mammal |


---

|  |  |
| --- | --- |
| Location | chromosome 9, 9q22.3 (Homo sapiens) |


---

|  |  |
| --- | --- |
